# Taking a molecular motor for a spin: helicase mechanism studied by spin labeling and PELDOR

**DOI:** 10.1093/nar/gkv1373

**Published:** 2015-12-10

**Authors:** Diana Constantinescu-Aruxandei, Biljana Petrovic-Stojanovska, Olav Schiemann, James H. Naismith, Malcolm F. White

**Affiliations:** 1 Biomedical Sciences Research Complex, University of St Andrews, Fife KY16 9ST, UK; 2 Institute of Physical and Theoretical Chemistry, University of Bonn, Wegelerstrasse 12, 53115 Bonn, Germany

## Abstract

The complex molecular motions central to the functions of helicases have long attracted attention. Protein crystallography has provided transformative insights into these dynamic conformational changes, however important questions about the true nature of helicase configurations during the catalytic cycle remain. Using pulsed EPR (PELDOR or DEER) to measure interdomain distances in solution, we have examined two representative helicases: PcrA from superfamily 1 and XPD from superfamily 2. The data show that PcrA is a dynamic structure with domain movements that correlate with particular functional states, confirming and extending the information gleaned from crystal structures and other techniques. XPD in contrast is shown to be a rigid protein with almost no conformational changes resulting from nucleotide or DNA binding, which is well described by static crystal structures. Our results highlight the complimentary nature of PELDOR to crystallography and the power of its precision in understanding the conformational changes relevant to helicase function.

## INTRODUCTION

Helicases are molecular motors that utilize the energy of adenosine triphosphate (ATP) binding and hydrolysis to drive cyclical protein conformational changes, propelling them along nucleic acids with a specific polarity. In addition to this translocation activity, helicases destabilize and hence unwind double-stranded nucleic acids by active or passive means. This nucleic acid remodeling activity is essential in all the cellular pathways that involve the manipulation of DNA or RNA including replication, recombination, repair, transcription and translation (1). Helicases have been classified into six Superfamilies (SF1–6) on the basis of their structure and conserved sequence motifs and also as subtype A or B for 3′-5′ or 5′-3′ polarity, respectively (2).

SF1 and SF2 helicases constitute a diverse group of enzymes with many different cellular functions. All share a catalytic core consisting of two ‘motor’ domains with a RecA-like fold that come together to form a binding site for ATP. In addition to the conserved core, SF1 and SF2 helicases sport a diverse array of N- and C-terminal extensions and inserted domains that define their specific physical and functional interactions with nucleic acids and other proteins. Three distinct classes of SF1 and 10 classes of SF2 helicases have been defined (3).

**Figure 1.**
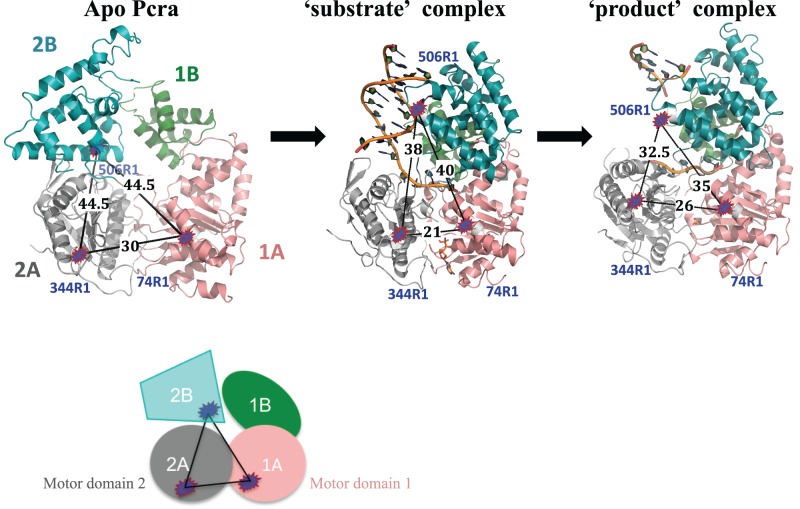
The main simulated distances (black) of PcrA expressed in Å between the spin labels for the cysteine pairs between specific domains: 1A–2A (74R1–344R1), 1A–2B (74R1–506R1) and 2A–2B (344R1–506R1). The spin label conformational distributions simulated with MtsslWizard are represented as blue–red shapes. The domains are colored salmon (1A), gray (2A), green (1B) and teal (2B). The apo PcrA represents unbound protein, ‘substrate complex’ is PcrA + DNA + AMP–PNP and ‘product’ complex is PcrA + DNA + SO_4_^2−^. The figure below is a simplified representation of the labeled protein.

X-ray crystallography has provided many of the key insights into helicase structure and mechanism. Early investigations of the SF1A UvrD/Rep family (PcrA, Rep, UvrD helicases) revealed the presence of two motor domains (named 1A and 2A), each harboring an inserted accessory domain (1B and 2B, respectively) (4,5). In complex with a double stranded DNA (dsDNA) having a (dT)_7_ single stranded DNA (ssDNA) overhang, and SO_4_^2−^ (‘product’ complex), the 2B domain of PcrA was observed to rotate by ∼160° across the top of the motor domains, generating a dsDNA-binding interface; identified as the ‘closed’ structure of PcrA, with the apo conformation representing the ‘open’ structure (6). Crystals obtained in the presence of both DNA and the non-hydrolysable ATP analog AMP–PNP (‘substrate’ complex) showed that the motor domains tightened around the nucleotide, with concomitant manipulation of the bound DNA (6). These structures (Figure 1) were interpreted as ‘snapshots’ of the helicase reaction pathway leading to the proposal of the ‘inchworm’ mechanism for DNA translocation by helicases driven by ATP binding and hydrolysis. Subsequently, a range of structures of the related SF1A helicase UvrD broadly confirmed these observations (7,8). Nevertheless, using intramolecular fluorescence resonance energy transfer (FRET) of UvrD bound to 3′-ssDNA/dsDNA junctions, with ssDNA between 10 and 40 nt, it was found that the fully closed conformation was not highly populated (8). The 2B domain seems to adopt many intermediate positions between completely open and completely closed that are influenced by salt, DNA and nucleotide binding. Addition of ATPγS or ADP^.^MgF_3_ (which should mimic an ADP-inorganic phosphate intermediate) induced a more closed structure compared with the binary UvrD–dsDNA complex (8).

PcrA was found to translocate and induce repetitive looping on the 5′-tail of a partial duplex or a fork DNA, an activity that would seem to require tight anchoring of the 2B domain to the duplex junction (9). The data indicated that the 2B orientation during looping may differ from that in the crystal structure, changing from closed to open at the initial stage of DNA binding and maintaining this conformation during translocation. Single molecule FRET studies found the 2B domain of Rep to be mostly in a closed state when bound to a DNA duplex with a 3′ tail, in agreement with the orientation observed in the crystal structure of PcrA bound to a similar DNA (10). Domain 2B of Rep seems to close gradually as the helicase approaches a duplex DNA junction during ssDNA translocation (11).

There thus remains some uncertainty on whether the crystal structures of PcrA, UvrD and other helicases fully represent true conformational intermediates that exist in solution during helicase activity, rather than inactive or dead-end complexes that have been trapped by crystallization (10,12). The issue is further complicated by several studies that have suggested these helicases unwind DNA only when present in dimeric/oligomeric form (13–21) or together with accessory proteins (22–26), although monomers can efficiently translocate along ssDNA (14,16,19). It has been proposed that the active form of UvrD is a dimer that is pre-assembled in solution in absence of DNA (17), however, all crystal structures published to date are monomeric. Domain 2B has been proposed to have a regulatory role, modulating helicase activity (14,27).

A recent study demonstrated DNA unwinding by monomeric UvrD accompanied by conformational changes of domain 2B via optical trapping and single-molecule FRET (28). It was found that the unwinding activity occurs with a closed conformation although less than 20 bp were unwound (so-called ‘frustrated’ unwinding). The monomer switches strands leading to duplex re-zipping, (observed previously (29)) and this is suspected to prevent long-distance unwinding. This switching occurs with movement of 2B and opening of the conformation. Dimeric protein unwinds longer distances indicating that interactions between monomers in the dimer prevent this strand switching. Cross-linking the 2B domain in the closed state yields a monomer that unwinds thousands of base pairs (30). These observations support the idea that maintenance of the closed conformation is key to long distance unwinding.

Much less structural information is available for the SF2B helicases in general and the helicase XPD (xeroderma pigmentosum complementation group D) in particular. XPD is part of the 10-subunit transcription factor IIH (TFIIH) in eukaryotes, which functions both in transcription initiation and nucleotide excision repair (NER), although the helicase activity of XPD is dispensable for the former (31,32). The archaeal orthologs of XPD are monomeric in solution and have proven more amenable to study with four apo crystal structures (PDB: 2vsf; PDB: 3crv; PDB: 3crw; and PDB: 2vl7) of archaeal XPD homologs reported (33–35). XPD has four domains: two RecA-like motor domains that form the motor core (named HD1 and HD2) and two auxiliary domains (4FeS domain and Arch domain) that are inserted in HD1. The 4FeS domain is stabilized by a 4Fe-4S cluster and is conserved in a family of eukaryotic SF2B helicases (36).

The crystal structure of XPD from *Thermoplasma acidophilum* (TaXPD) in complex with a 4 nt DNA bound in a cleft in HD2 (PDB: 4a15) represents the only structure of an SF2B helicase bound to DNA (37). Although the structure confirmed the predicted polarity of the translocated strand, it did not disclose how the DNA binds to the rest of the protein, nor the conformational changes that accompany binding. The commonly accepted helicase mechanism of XPD involves the passage of the translocated strand through the pore formed by Arch, 4FeS and HD1 domains (33–35,37,38). Such a model suggests that the XPD region interacting with the translocated strand extends beyond the canonical DNA binding site, with a second binding site between HD1 and 4FeS domains (38–40). As all crystal structures show the central pore to be topologically closed through contacts between the Arch domain and 4FeS domain, XPD needs to undergo a conformational change that opens the pore to allow binding to ssDNA within a repair bubble (41).

Movement of the Arch domain has been followed by quenching of a covalently attached Cy3 fluorophore by the 4FeS cluster in a single molecule system (42). Transitions between the closed state corresponding to the crystal structure and what was proposed to be the open state were observed both in absence and presence of DNA. The dwell times of the open and closed conformations were fitted to a double and a three exponential function, respectively. Interestingly, the fast components of the multi-exponential dwell time distributions represented 75% of the total amplitude for both the open and closed states. The conformational transitions were slow (subsecond to tens of seconds time scale) and the weighted mean lifetime of the closed conformation was 3-fold longer than that of the open state. This is consistent with the observation in crystal structures of the Arch domain in a closed conformation. The most intriguing findings were that 70% of the DNA binding events were initiated when XPD was in the Arch closed conformation and DNA binding did not change the distribution of protein conformations.

The difficulty inherent in studying the conformational changes of helicases during different stages in the reaction cycle still presents a formidable barrier to a definitive understanding of helicase operation. We have used site directed spin labeling (SDSL) and pulsed EPR (PELDOR) (43) to investigate the conformations of PcrA and XPD, as representatives of two important helicase families, at different stages in their reaction cycles, in frozen solution. We demonstrate that PELDOR reliably detects and quantifies small conformational changes. In case of PcrA, we were able to monitor not only the larger conformational changes of domain 2B induced by DNA, but also the smaller ones between the motor domains due to nucleotide binding. In contrast XPD is revealed to be a much more rigid protein without significant conformational changes under these experimental conditions, highlighting the diversity of helicase mechanisms.

## MATERIALS AND METHODS

### PcrA expression, purification and site directed mutagenesis

The recombinant plasmid pET22b with ampicillin resistance, which contains the sequence of PcrA from *Bacillus stearothermophilus* was a gift from Mark S. Dillingham. Pairs of cysteine residues for spin labeling were introduced by site directed mutagenesis (the sequences of mutagenic oligos are available from the corresponding author on request). The wild-type (wt) and all the mutants were transformed in *Escherichia coli* (*E. coli*) BL21 (DE3) cells. The cells were grown in LB (Luria-Bertani) medium with 100 μg/ml ampicillin at 37°C. The protein expression was induced by the addition of 1 mM isopropyl-b-D-thiogalactoside (IPTG) and the cells were further grown at 37°C for 3 h. The cells were harvested, resuspended and lysed by sonication in lysis buffer (50 mM Tris/HCl pH 7.5, 200 mM NaCl, 1 mM DTT, 1 mM EDTA and protease cocktail), followed by centrifugation at 40 000 x *g* at 4°C for 40 min.

The protein was bound to a 5 ml Heparin column (GE Healthcare) equilibrated with Buffer A (50 mM Tris/HCl pH 7.5, 100 mM NaCl, 1 mM DTT and 1 mM EDTA) and eluted with a linear gradient of 1 M NaCl in the same buffer. The fractions containing the protein were identified by sodium dodecyl sulphate-polyacrylamide gel electrophoresis (SDS-PAGE), pooled and further purified on a HiLoad 26/60 Superdex 200 column (GE Healthcare) equilibrated with gel-filtration (GF) buffer (50 mM Tris/HCl pH 7.5, 500 mM NaCl, 1 mM EDTA). The protein-containing fractions were examined by SDS-PAGE and the pure fractions were pooled and concentrated.

### TaXPD expression, purification and site directed mutagenesis

A synthetic gene encoding TaXPD in which the three native cysteines (not ligated to the iron cluster) were mutated to alanine was designed and purchased (DNA2.0, USA). The gene was supplied in the pJexpress401 vector with kanamycin resistance and a TEV-cleavable N-terminal 6-histidine tag for affinity purification was included. The ‘no cysteine native’ XPD, as well as mutants of this gene into which specific cysteine residues were introduced, were transformed in *E. coli* Rosetta cells (the sequences of mutagenic oligos are available from the corresponding author on request). The cells were grown in LB medium supplemented with 35 μg/ml kanamycin at 37°C. When OD_600nm_ reached 0.8–1, the temperature was lowered to 28°C and the protein expression was induced with 0.25 mM IPTG overnight. The cells were harvested (15 000 x *g*, 15 min, 4°C), resuspended and lysed by sonication in ice-cooled lysis buffer (20 mM Tris/HCl pH 7.5, 500 mM NaCl, 10 mM imidazole and one EDTA-free protease-inhibitor tablet), followed by centrifugation at 40 000 x *g* at 4°C for 40 min. After passage through a 0.45 μm filter, the supernatant was loaded on a Ni-column equilibrated with the lysis buffer and the column was washed with buffer A (20 mM Tris/HCl pH 7.5, 500 mM NaCl, 30 mM imidazole) until the absorption reached the baseline. The proteins were eluted with an imidazole gradient running from 30 to 500 mM, with protein generally eluting at around 170 mM imidazole. The fractions containing the protein were identified by SDS-PAGE, pooled and dialysed for 2 h in 20 mM Tris pH 7.5, 500 mM NaCl, 1 mM DTT. The His-tag was cleaved overnight by adding 0.1× TEV protease in fresh buffer. Next day, the protein was loaded again on a Ni-column equilibrated with the lysis buffer (without the protease inhibitor tablet) and washed with the same buffer until the absorption reached the baseline. The cleaved protein, which does not bind to the column, was collected in the flow-through. The protein was concentrated down to 5–6 ml, spin-labeled and loaded on to a HiLoad 26/60 Superdex 200 gel filtration column (GE Healthcare) equilibrated with gel-filtration (GF) buffer (20 mM Tris/HCl pH 7.5, 200 mM NaCl). The protein-containing fractions were verified for purity by SDS-PAGE and the pure fractions were pooled and concentrated.

### Fluorimetric helicase kinetic assay to examine XPD inhibition by nucleotide analogs

Binding of ATP analogs to XPD was confirmed by measuring their ability to inhibit the helicase activity of XPD in a continuous fluorescent assay, adapted from (37). In brief, a forked DNA species was assembled by annealing the following two oligonucleotides (IDT), one with a dabcyl modification on the 3′-end (5′- AGC TAC CAT GCC TGC ACG AAT TAA GCA ATT CGT AAT CAT GGT CAT AGC T-3′-dabcyl) and the other with a Cy3 label at the 5′-end (Cy3–5′- AGC TAT GAC CAT GAT TAC GAA TTG CTT GGA ATC CTG ACG AAC TGT AG-3′). In the duplex, Cy3 fluorescence is quenched by the dabcyl moiety that resides in close vicinity. This quenching is removed upon unwinding by XPD. DNA (50 nM) was unwound in helicase buffer (20 mM MES pH 6.4, 0.1 mg/ml BSA (bovine serum albumin), 1 mM MgCl_2_ and 1 mM ATP) with a final total volume of 150 μl and fluorescence change over time was monitored in a Cary Eclipse fluorescence spectrophotometer (Varian), at 20°C. The data are shown in Supplementary Figure S12.

### Site directed spin labeling (SDSL) of PcrA

Spin labeling was carried out by mixing 20 μM PcrA with 100 μM MTSSL in 10 mM TES buffer pH 7.4 and 500 mM NaCl with a total volume of 2.5 ml at 4°C for 1 h. The excess spin label was removed using a Sephadex G-25 mini column (GE Healthcare) according to the manufacturer's instructions. Mass spectrometry was used to confirm the labeling. The spin-labeled mutants are denoted as xR1, where x represents the residue number. Proteins were exchanged in D_2_O (Aldrich) buffer with the same composition as the labeling buffer, by serial dilutions and concentrations with a centricon.

### SDSL of TaXPD

Cysteine mutants of XPD were observed to be prone to aggregation; labeling the cysteines before gel filtration significantly reduced the aggregation. After the second Ni column, the protein was concentrated down to 5–6 ml and incubated with approximately 10× excess of MTSSL for one hour at 4°C before loading on the gel filtration column.

### Sample preparation for PELDOR

All oligodeoxynucleotides were purchased from IDT (Integrated DNA Technologies). The PcrA protein (70 μM) was mixed in the D_2_O buffer with ds/ssDNA and/or nucleotide. The following combinations were prepared for each mutant: PcrA alone, PcrA + AMP–PNP/MgCl_2_, PcrA + ADP/MgCl_2_, PcrA + dsDNA, PcrA + dsDNA and AMP–PNP/MgCl_2_ and PcrA + dsDNA and ADP/MgCl_2_. The DNA used forms a hairpin structure with an 11 bp duplex and a 3′-(dT)_10_ overhang (5′-CGA GCA CTG CTT TAG CAG TGC TCG TTT TTT TTT T-3′). It should be noted that this is not the same sequence used in the original crystallographic studies of PcrA. The DNA concentration was kept constant at 150 μM in all samples. The nucleotide was typically added in a 10-fold excess over protein and MgCl_2_ had 1 mM final concentration. The nucleotide-triphosphate will be used as a general term when referring to either ATP or its non-hydrolysable analogs (AMP–PNP and ATPγS). Deuterated ethylene glycol (EG-d6) was added as a final 5% (v/v) concentration at the end.

In case of XPD, the protein concentration was typically 40–50 μM, the DNA concentration approximately double and the nucleotide/MgCl_2_ concentration was 1 mM, unless otherwise indicated. The ssDNA was a (dT)_16_ oligonucleotide; the bubble DNA (bDNA) was a 18-mer helix on each side of a (dT)_20_ bubble, with the following forward sequence:

5′- GCG TAG TAT GCT CAG CGG TTT TTT TTT TTT TTT TTT TTC GCC AGC GTT TCC CAG TC-3′
and the reverse sequence:
5′- GAC TGG GAA ACG CTG GCG TTT TTT TTT TTT TTT TTT TT C CGC TGA GCA TAC TAC GC-3′.

All oligonucleotides were purchased from IDT (Integrated DNA Technologies). All samples were prepared in 20 mM Tris buffer pH 7.5. The EG-d6 concentration was typically 30–40% (v/v). As a control, other concentrations (10 and 50%) were also tested, with no significant influence on the distance distribution (Supplementary Figure S8). The only significant (and expected) effect was on the degree of instantaneous diffusion (aggregation); the higher the EG-d6 concentration, the lower the aggregation (observed in the background decay) due to less ice formation on freezing the sample.

### PELDOR data collection and analysis

All PELDOR experiments on PcrA and some on XPD were performed using a Bruker ELEXSYS E580 spectrometer operating at X-band (9.4 GHz), having an MD4 (PcrA) or MD5 (XPD) dielectric ring resonator and a Bruker 400U second microwave source unit. Pulses are amplified by a pulsed travelling wave tube (TWT) amplifier with a nominal output of 1 kW. All samples were measured at 50 K with an overcoupled resonator (quality factor Q ∼100). The video bandwidth was set at 20 MHz. The four-pulse, dead-time free, PELDOR sequence was used (44); the pump pulse frequency was positioned at the maximum of the nitroxide spectrum and the frequency of the observer pulses was increased by 80 MHz. The π/2 pulse length for the observer sequence was set to 16 ns and the pump π-pulse was typically 16–20 ns. Since a deuterated solvent was used, the first inter-pulse delay in the PELDOR sequence was set to 380 ns, which corresponds to a blind spot of the deuterium modulation. Such choice is based on the fact that at this value most of the deuterium contribution via the Electron Spin-Echo Envelope Modulation (ESEEM) effect is suppressed. The first interpulse delay was also varied eight times by 8 ns each time in order to average the proton nuclear modulation. Two-step phase cycling was used to eliminate receiver offsets. The experiment repetition time was 3 or 4 ms and 50 shots were used at each time point. The total experiment time was typically 24–48 h, depending on the signal to noise.

Some XPD measurements were performed using a Bruker ELEXSYS E580 operating at Q-band (34 GHz, 150 W nominal output of TWT amplifier, cylindrical resonator ER 5106QT-2w). The pump pulse frequency was positioned at the maximum of the nitroxide spectrum and the frequency of the observer pulses was decreased by 80 MHz. The π/2 pulse length for the observer sequence was set to 12 ns and the pump π-pulse was typically 12 or 14 ns. The total experimental time was typically 1–2 h, due to the higher sensitivity of Q-band.

The experimentally obtained time domain traces were analysed with DeerAnalysis 2013 (45). The unwanted intermolecular couplings were removed by background decay correction. Generally, a homogeneous 3D spin distribution was used for the correction. The starting time for the background fit was optimized to give the best fit Pake pattern in the Fourier transformed data and the lowest rmsd background fit. This was followed by Tikhonov regularization (45,46) in order to simulate time trace data and give rise to distance distributions, P(r), of different peak width depending on the regularization factor, α. The α value was chosen based on a calculated L-curve (shown in Supplementary Information) but also by inspecting the quality of the fit. In general, this value was at the inflection of the L curve, which provides the best compromise between smoothness (artefact suppression) and fit to the experimental data. Also, the real distances were checked with the validation tool available in DeerAnalysis (see the example for XPD 122R1–306R1 in Supplementary Figure S10).

### Prediction of distance distributions based on the crystal structures

The MtsslWizard plugin (47,48) for the software package Pymol was used for *in silico* spin labeling, rotamer conformation searching and distance measurements. The vdW cutoff was either 2.2 Å or 3.4 Å with 0 clashes allowed. In some cases, it was observed that the distance distribution width (mainly) and to a less extend the main distance depended on the cutoff. The difference in the main distance was nevertheless relatively small, within few Å. The cutoff that gave the best similarity to the experimental data was finally chosen. The atomic coordinates used for modelling were as follows: 1pjr (apo PcrA), 2pjr (the ‘product’ complex, PcrA + DNA and SO_4_^2−^), 3pjr (the ‘substrate’ complex, PcrA + DNA and AMP–PNP), 1qhh (PcrA + AMP–PNP), 1qhg (PcrA K37A + AMP–PNP), 2vsf (TaXPD alone) and 4a15 (TaXPD + 4 nt DNA). The distance distributions were generated by binning the data into 0.5–1 Å bins.

## RESULTS

### PcrA

**Figure 2.**
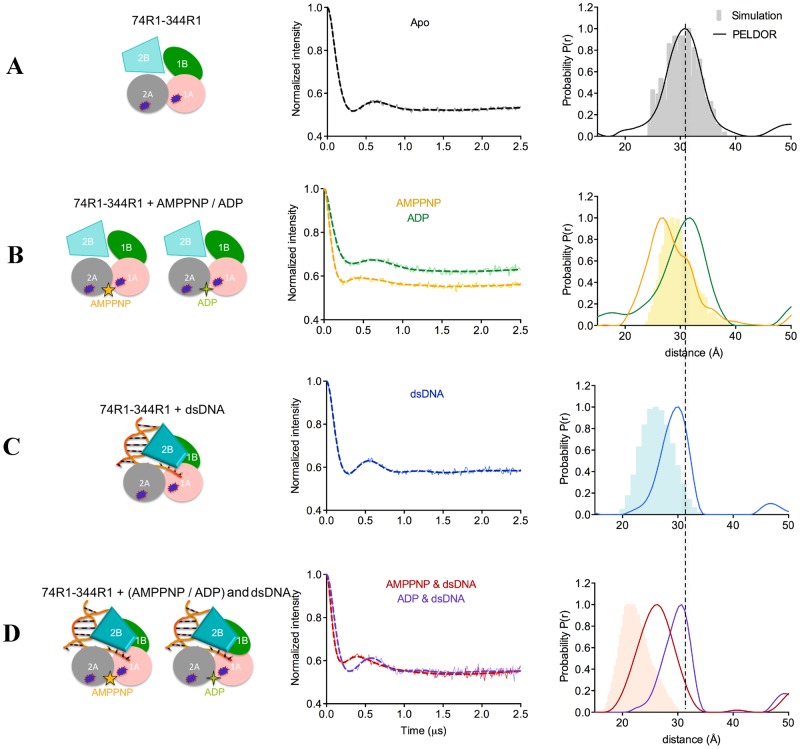
**(A-D)** PELDOR data of PcrA 1A–2A (74R1–344R1). (Left) Diagrams representing PcrA in the specified binding states, with the attached spin labels shown as dark blue shapes. (Middle) Background-corrected time traces (continuous lines) and the most appropriate simulations (dotted lines) of the experimental data based on the L-curve (Supplementary Figure S2B in Supplementary Information). The raw time traces are shown in Supplementary Figure S2A. (Right) Tikhonov derived distance distributions (continuous lines) and model-derived MtsslWizard simulations (gray (free protein), light orange (AMP–PNP), light blue (DNA) and light salmon (AMP–PNP and DNA) shapes).

**Figure 3.**
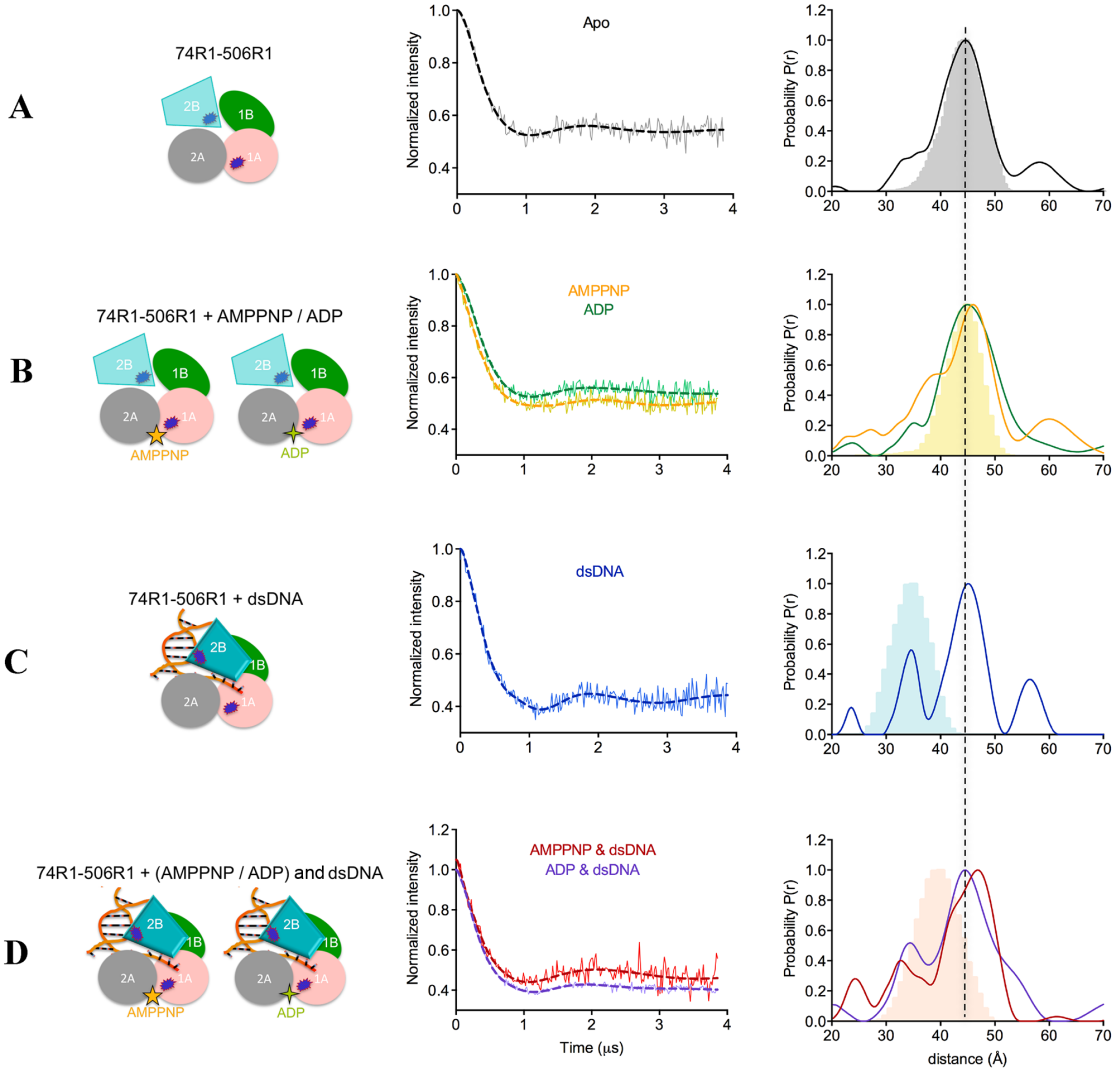
**(A-D)** PELDOR data of PcrA 1A–2B (74R1–506R1). (Left) diagrams representing PcrA in the specified binding states, with the attached spin labels shown as dark blue shapes. (Middle) Background-corrected time traces (continuous lines) and the most appropriate simulations (dotted lines) of the experimental data based on the L-curve (Supplementary Figure S3B in Supplementary Information). The raw time traces are shown in Supplementary Figure S3A. (Right) Tikhonov derived distance distributions (continuous lines) and model-derived MtsslWizard simulations (gray (free protein), light orange (AMP–PNP), light blue (DNA) and light salmon (AMP–PNP and DNA) shapes). The left diagrams represent PcrA in the specified binding states, with the attached spin labels shown as dark blue shapes.

**Figure 4.**
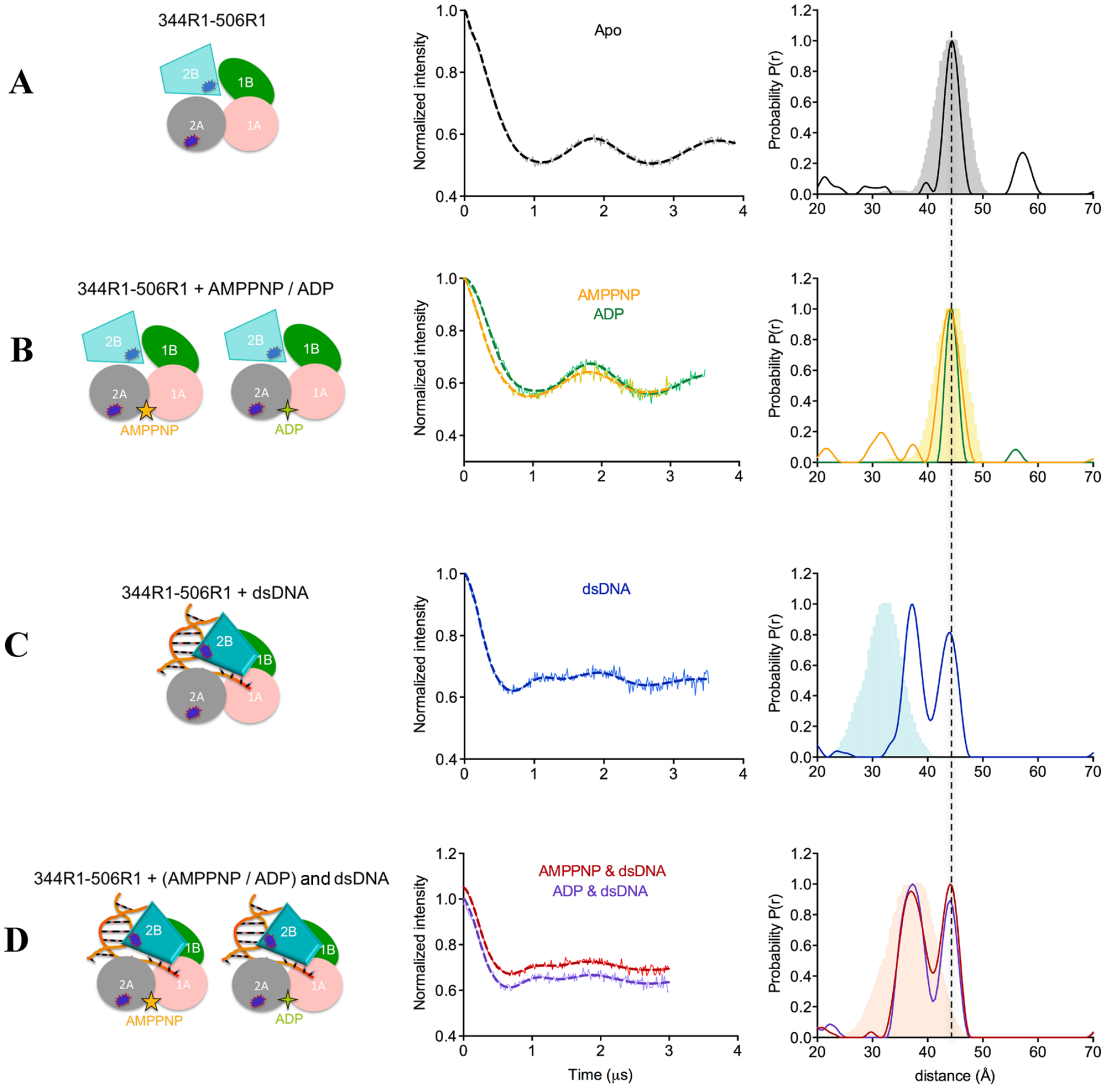
**(A-D)** PELDOR data of PcrA 2A–2B (344R1–506R1). (Left) diagrams representing PcrA in the specified binding states, with the attached spin labels shown as dark blue shapes. (Middle) Background-corrected time traces (continuous lines) and the most appropriate simulations (dotted lines) of the experimental data based on the L-curve (Supplementary Figure S4B in Supplementary Information). The raw time traces can be found in Supplementary Figure S4A. (Right) Tikhonov derived distance distributions (continuous lines) and model-derived MtsslWizard simulations (gray (free protein), light orange (AMP–PNP), light blue (DNA) and light salmon (AMP–PNP and DNA) shapes). The left diagrams represent PcrA in the specified binding states, with the attached spin labels shown as dark blue shapes.

Three double cysteine mutants of PcrA were constructed to monitor distances between domains 1A, 2A and 2B: E74C (1A domain)–V344C (2A domain), E74C (1A domain)–Q506C (2B domain) and V344C (2A domain)–Q506C (2B domain). In each case, the purified variants were labeled with MTSSL (the labeled cysteines are denoted here 74R1, 344R1 and 506R1) and the incorporation of the spin label was confirmed by mass spectrometry. Moreover, based on the PELDOR modulation depth, the labeling degree was at least 90% for all double mutants. Figure 1 presents the three labeled cysteine mutants for three binding states of PcrA based on the respective crystal structures: apo protein, ‘substrate’ complex (DNA and AMP–PNP) and ‘product’ complex (DNA and SO_4_^2−^), showing the predicted distances in Angstroms between labels based on the known crystal structures. The *in-silico* MtsslWizard simulations of all spin labels are shown in Supplementary Figure S1. For the free PcrA, the experimental distance distributions determined by PELDOR are in very good agreement with the predicted ones (Figures 2A, 3A and 4A, Table 1), with just small variations in distribution width for 74R1–344R1 (Figure 3A) and 344R1–506R1 (Figure 4A). This confirmed that the labeling did not affect the overall structure of PcrA.

**Table 1.** Summary of the main distances of PcrA in different binding states from MtsslWizard and PELDOR

Mutant	Apo	AMP–PNP	ADP	DNA	DNA and AMP–PNP	DNA and ADP
E74-V344 (1A-2A)	30/30.7	29/26.5 and 30.7 (1:0.7)	/31.7	26/30	21/26	/30.6
E74-Q506 (1A-2B)	44.5/35 and 45 (0.2:1)	45/39.5 and 46 (0.5:1)	/45	35/35 and 45 (0.5:1)	40/33 and 46 (0.4:1)	/34 and 45 (0.5:1)
V344-Q506 (2A-2B)	44.5/44.4	44.5/44.1	/44.4	32.5/37.3 and 44.1 (1:0.7)	38/36.7 and 44.1 (1:1)	/36.7 and 44.4 (1:1)

The values on the left side of the slash line represent the simulated distances based on the crystal structures; the values on the right side are the experimental values from PELDOR. All distances are expressed in Å. In case of two distances, the ratio between them is shown in brackets.

### Effect of nucleotide binding on PcrA conformation

The two motor domains of SF1A helicases are expected to move closer together when nucleotide triphosphate binds (49). There are two published crystal structures of PcrA with AMP–PNP, one of the WT protein (50) (PDB: 1qhh) and one of the K37A variant (50) (PDB: 1qhg). Both structures were obtained by soaking the nucleotide into PcrA crystals and no significant conformational changes between the motor domains was observed. Accordingly, the predicted distance between the motor domains for the apo and AMP–PNP-bound protein (Figure 2A and B, respectively, Table 1) are essentially unchanged (the small difference may arise from the slightly different orientations of the label). The crystal structure predicts also no change in the configuration of the motor domains with respect to domain 2B (Figure 3B versus 3A and Figure 4B versus 4A).

The distance between the motor domains (1A and 2A) in the apo conformation obtained by PELDOR was measured as 30.7 Å, in agreement with the predicted distance based on the crystal structure (30.0 Å, Figures 1 and 2A, Table 1). Upon addition of AMP–PNP, PELDOR data reports a main distance of 26.5 Å which we assign as the nucleotide bound form and a shoulder at around 30.7 Å suggesting some of the apo form remains or an equilibrium exists between two conformers (Figure 2B). The distance change (4 Å) upon nucleotide binding was not observed in the crystal structure.

In contrast to AMP–PNP, ADP binding slightly increased the distance between the motor domains by 1 Å, to 31.7 Å and it induced a broader distance distribution compared to the apo state (Figure 2B and Table 1).

In the apo state, the distance distribution between domains 1A and 2B was much broader than the distance distribution between 2A and 2B (Figures 3A and 4A, respectively). The PELDOR data for the 1A–2B distance in the apo form exhibits a shoulder corresponding to a shorter distance (Figure 3A). Although ADP binding had little affect on this distribution, binding of AMP–PNP broadened this distance distribution further (Figure 3B). Neither AMP–PNP nor ADP binding affected the distance between 2A and 2B (Figure 4B).

From these PELDOR data we conclude that motor domain 1A is flexible relative to motor domain 2A and domain 2B in absence of nucleotide and that binding of nucleotide-triphosphate to PcrA brings domain 1A closer to 2A and 2B. ADP has very little effect upon the structure.

### Conformational changes of PcrA bound to DNA

Crystal structures have shown that DNA binding results in a swivelling of ∼160° of domain 2B across the top of the motor domains (Figure 1). Examination of the 1A–2B pair (74R1–506R1) in the crystal structure of the ‘product complex’ would predict that binding of DNA shortens the distance by 10 Å, from 44.5 to 35 Å. For the 2A–2B (344R1–506R1) pair, the distance would be shortened by ∼12 Å, from 44.5 to 32.5 Å (Figure 1, Table 1).

PELDOR measurements of the PcrA–DNA complex showed the appearance of a second shorter distance for both the 1A–2B and 2A–2B vectors (Figures 3C and 4C, respectively). The 1A–2B pair had a measured distance of 35 Å in agreement with the predicted one (Figure 3C, Table 1) whilst 2A–2B pair, the shorter experimental distance was 5 Å longer than that predicted from the crystal structure (Figure 4C, Table 1). However, the longer distance remained, indicating either that only a fraction of the protein was bound to DNA or that only a fraction of the DNA-bound protein adopts the crystal structure conformation (Figures 3C and 4C, Table 1).

The crystal structure of the ‘product complex’ indicates a change in the relative position of the motor domains when DNA binds; the 1A–2A pair (74R1–344R1) is predicted to shorten ∼4 Å compared with the apo protein. In contrast, the PELDOR data show that DNA did not have a significant effect on this distance, but that it reduced the flexibility of the motor domains (a narrower distribution with a peak at a 0.7 Å shorter distance of 30 Å (Figure 2C and Table 1)).

These data show that PELDOR confirms the movement of domain 2B when DNA binds but do not support the conclusion of the crystal structure that DNA profoundly alters the conformations of the motor domains.

### Conformational changes of PcrA in the ternary PcrA–nucleotide–DNA complexes

The crystal structure of the ternary PcrA–AMP–PNP–DNA complex (‘substrate’ complex) has a similar major conformational change of domain 2B observed in the PcrA–DNA complex. However, the predicted distances of the ternary structure are ∼5 Å longer than those predicted in the PcrA–DNA crystal structure for both 1A–2B (Figure 3D versus 3C) and 2A–2B pair (Figure 4D versus 4C), although all are shorter than the apo distances.

PELDOR showed the presence of distances that correlated with rotation of domain 2B and ‘closure’ of the structure. For 1A–2B (74R1–506R1) this distance (33 Å) was weakly populated compared to the apo distance and is in fact 7 Å shorter than that predicted (40 Å) by the ternary complex crystal (Figure 3D, Table 1)). However the distance is 2 Å shorter than the one measured for the PcrA–DNA complex (Table 1), indicating that AMP–PNP binding results in additional tightening of domains 1A and 2B. The distance distribution was very heterogeneous, most probably reflecting multiple conformational states of domains 1A and 2B in the ternary complex. PcrA 2A–2B (344R1–506R1) gave two distances with a ratio 1:1, indicating two distinct, equally populated conformations. This is a similar result to DNA alone, therefore AMP–PNP does not cause a significant change in the relative positions of domains 2A and 2B (Figure 4D versus 4C, Table 1). The shorter distance is in good agreement with the crystal structure.

The crystal structure shows a 5 Å tightening of the motor domains around AMP–PNP compared with the ‘product’ complex (Figure 2D versus 2C; Table 1). The PELDOR data report a 4 Å shorter distance compared with DNA alone (Figure 2D versus 2C; Table 1). The distance distribution between the two motor domains obtained from PELDOR (Figure 2D) is a single narrow peak at essentially the same shorter distance observed with AMP–PNP alone (Figure 2D versus 2B, Table 1). Thus, PELDOR data show a consistent tightening of the motor domain on binding nucleotide triphosphate, regardless of the presence of DNA. ADP added to the DNA complex resulted in no additional change in any of the three domain pairs (Figures 2D, 3D and 4D). Thus it appears that AMP–PNP alone can bring the motor domains closer together, while only DNA induces the movement of domain 2B. We did not detect any evidence of higher oligomeric states of PcrA in any of the PELDOR experiments.

### XPD

**Figure 5.**
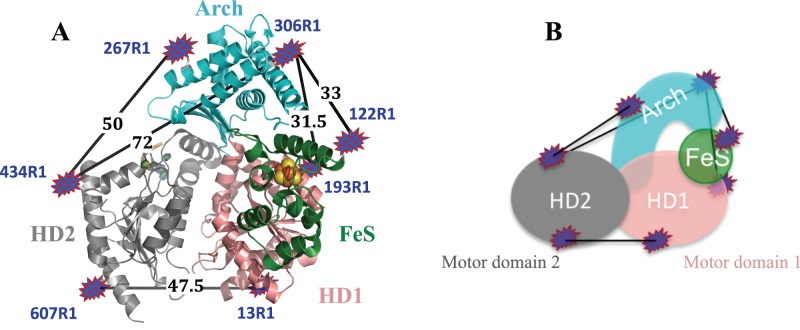
(**A**) The main simulated distances (black) of XPD expressed in Å between the spin labels for the cysteine pairs between specific domains: HD1–HD2 (13R1–607R1) (47.5 Å), 4FeS–Arch (122R1–306R1) (33 Å), HD1–Arch (193R1–306R1) (31.5 Å), Arch–HD2 (267R1–434R1 (50 Å) and 306R1–434R1 (72 Å)). The spin label conformational distributions simulated with MtsslWizard are represented as blue-red shapes. The domains are colored salmon (HD1), gray (HD2), green (4FeS domain) and cyan (Arch domain). (**B**) Simplified representation of the labeled protein.

For XPD, the four domains are denoted HD1, HD2, Arch and FeS (Figure 5). The following double cysteine mutants were created for XPD: D13C–N607C (HD1–HD2), A122C–Y306C (4FeS–Arch), E193C–Y306C (HD1–Arch), Y306C–D434C and S267C–D434C (Arch–HD2). Figure 5 presents the labeled cysteine mutants, showing the predicted distances between labels for the cysteine pairs measured. The PDB entry 4a15 was used for the most part as it includes the Q-motif, although it contains a 4-nt DNA. The differences in the predicted main distances between 4a15 and the apo structure (PDB: 2vsf) are typically less than 1 Å. In the case of the mutant pairs between the HD2 and Arch domains (where the 4 nt DNA binds), we used the predicted distances for both the apo and DNA complexes.

**Figure 6.**
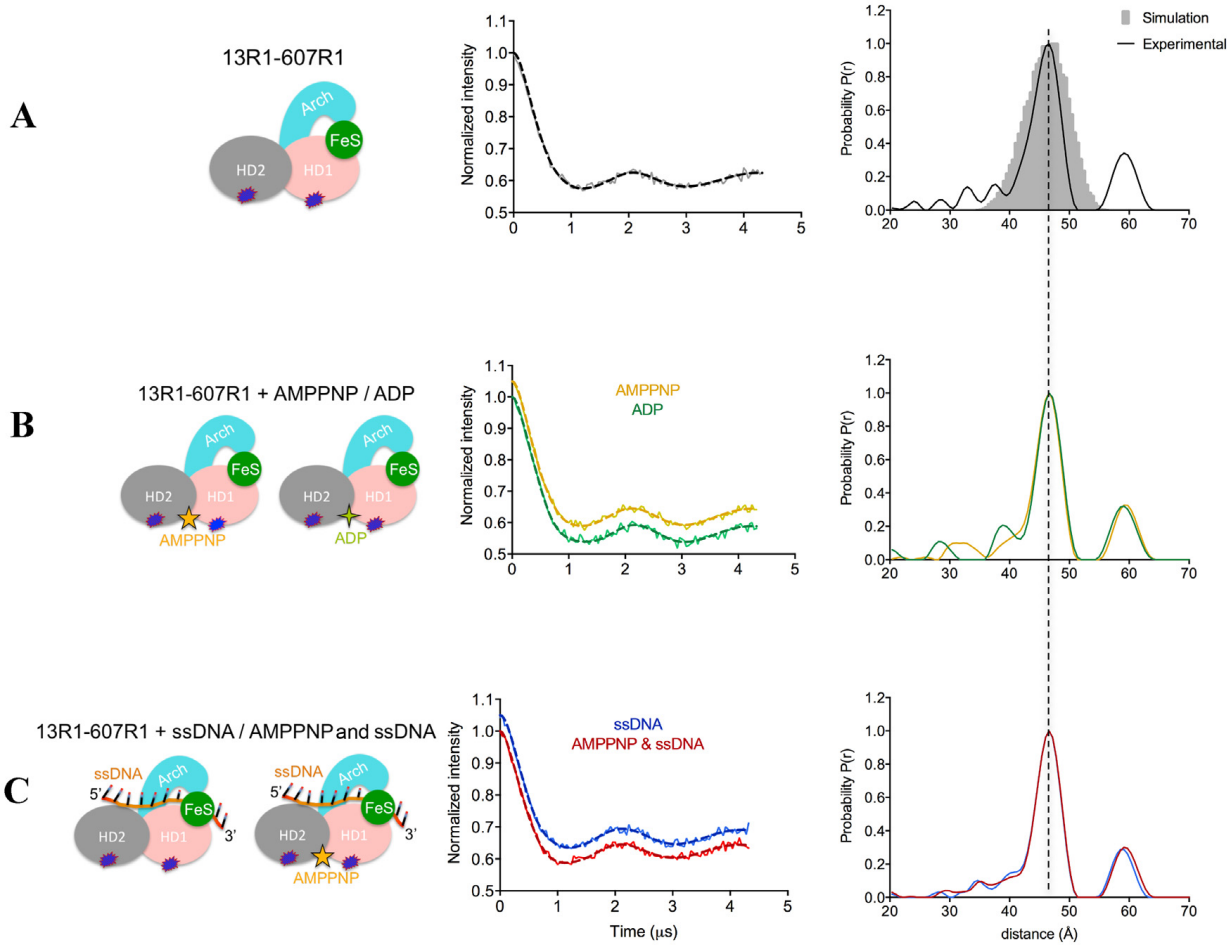
**(A-C)** PELDOR data of XPD HD1-HD2 (13R1–607R1). (Left) Diagrams representing XPD in the specified binding states, with the attached spin labels shown as dark blue shapes. (Middle) Background-corrected time traces (continuous lines) and the most appropriate simulations (dotted lines) of the experimental data of XPD HD1–HD2 based on the L-curve (Supplementary Figure S5B in Supplementary Information); the raw time traces are shown in Supplementary Figure S5A. (Right) Tikhonov derived distance distributions of apo XPD HD1–HD2 and XPD in the presence of different nucleotides/DNAs (continuous lines) compared with model-derived distance distribution (gray shape) based on crystal structure (PDB: 4a15).

**Figure 7.**
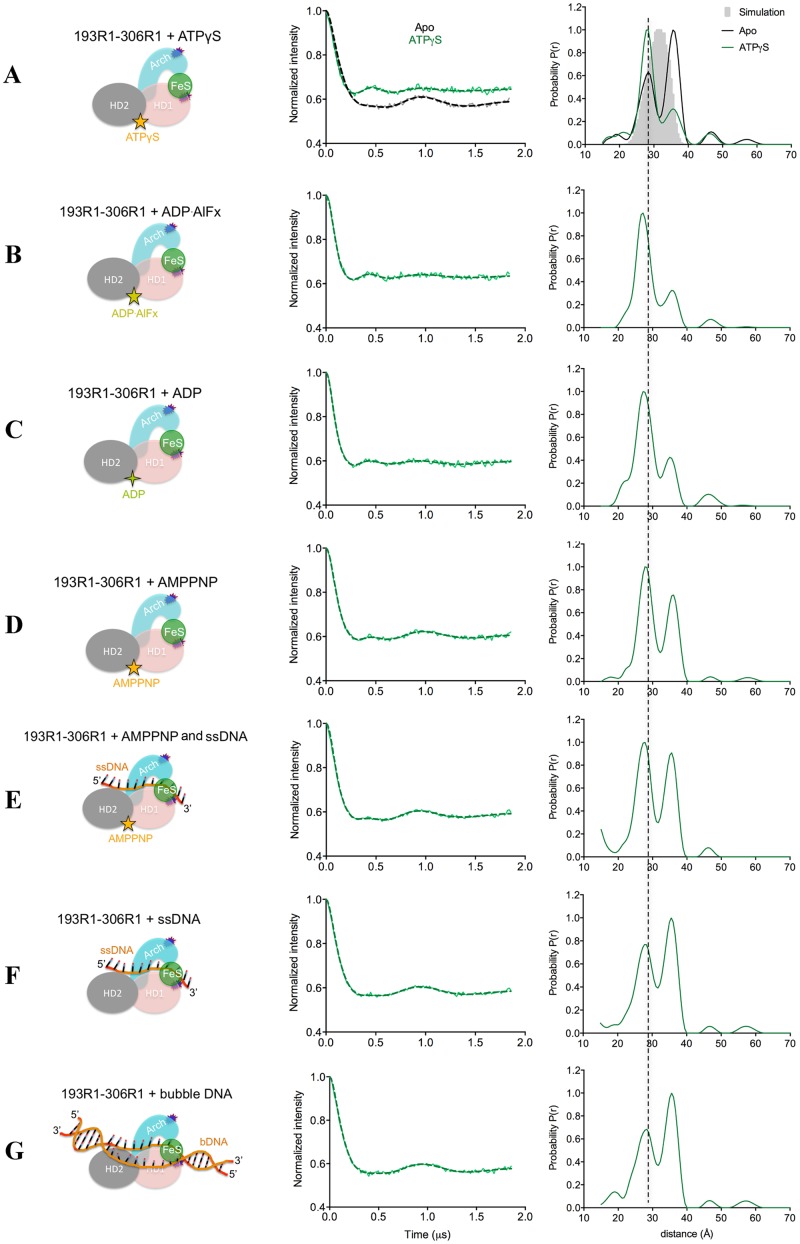
**(A-G)** PELDOR data of XPD HD1-Arch (193R1–306R1). (Left) Diagrams representing XPD in the specified binding states, with the attached spin labels shown as dark blue shapes. (Middle) Background-corrected time traces (continuous lines) and the most appropriate simulations (dotted lines) of the experimental data based on the L-curve (Supplementary Figure S6B in Supplementary Information); the raw time traces are shown in Supplementary Figure S6A. (Right) Tikhonov derived distance distributions of apo XPD HD1–Arch and XPD in the presence of different nucleotides/DNAs (continuous lines), compared with model-derived distance distribution (gray shape) based on crystal structure (PDB: 4a15).

**Figure 8.**
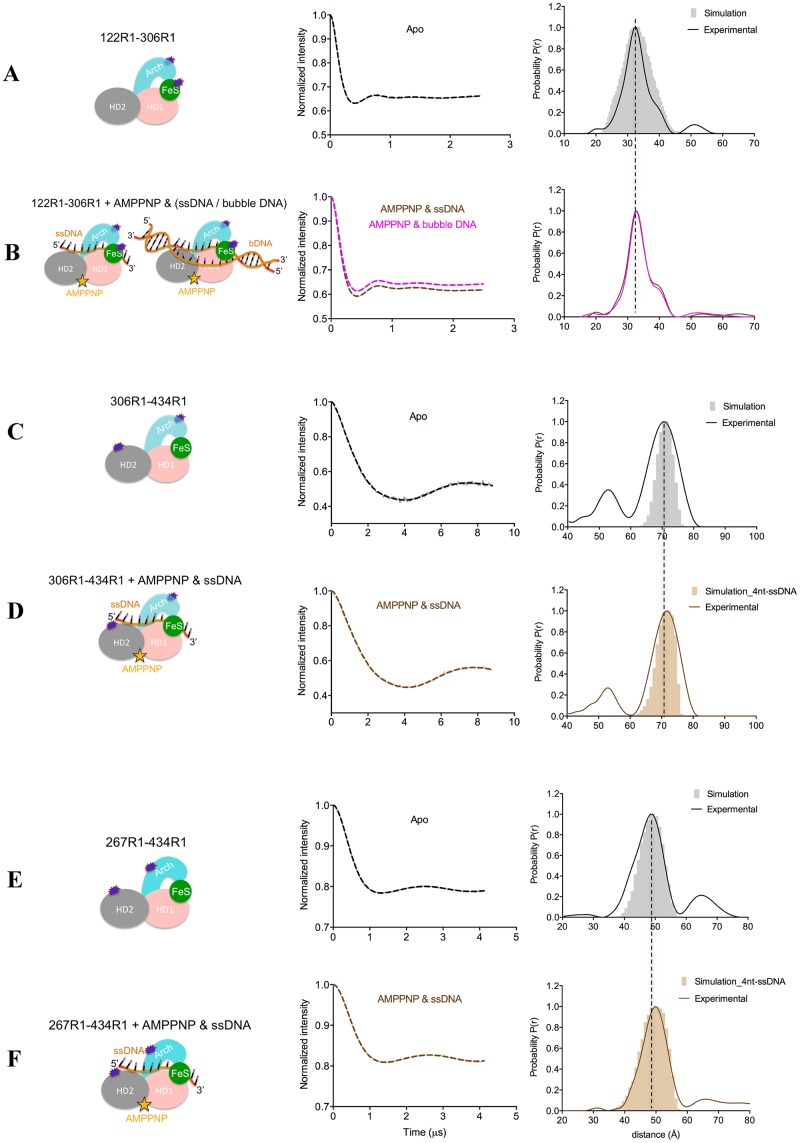
PELDOR data of XPD 4FeS-Arch (122R1–306R1) **(A, B)**, Arch-HD2 (306R1-434R1) **(C, D)**, and Arch-HD2 (267R1-434R1) **(E, F)**. (Left) Diagrams representing XPD in the specified binding states, with the attached spin labels shown as dark blue shapes. (Middle) Background-corrected time traces (continuous lines) and the most appropriate simulations (dotted lines) of the experimental data based on the L-curve (Supplementary Figure S7 in Supplementary Information) for 122R1–306R1, 306R1–434R1 and 267R1–434R1; the raw time traces are shown in Supplementary Figure S7. (Right) Tikhonov derived distance distributions of apo XPD and XPD in the presence of AMP–PNP and ssDNA/bubble DNA (continuous lines) compared with model-derived distance distributions (gray and light brown shapes) based on crystal structures (PDB: 2vsf was used for the apo TaXPD 306R1–434R1 and 267R1–434R1).

All cysteine mutants were successfully labeled with MTSSL with the exception of C267, which had ∼50% labeling efficiency, based on mass spectrometry and the PELDOR modulation depth. Nevertheless, we were still able to obtain reliable measurements for the C267–C434 pair. All cysteine pairs gave time traces with good oscillations both before and after background subtraction (Figures 6–8 and Supplementary Figure S5–S7) and narrow distance distributions, indicating rigid protein structures.

### Distance distributions in the apo protein

The experimental distance distribution between the two motor domains (HD1 and HD2) of the apo state was at 47 Å matching that predicted from the crystal structure (Figure 6A). Likewise the distance distributions of the two labeled cysteine pairs (306R1–434R1 and 267R1–434R1) that reported on the separation between the HD2 and Arch domains (Figure 8C–F, respectively) matched the expected distances very well, as did the distance distribution measured for the spin pair between the Arch and the 4FeS domains (32 Å). This distribution showed a shoulder around 40 Å with about 1/5 of the maximum intensity that was not predicted by the structure (Figure 8A). For the Arch–HD1 pair, there were two sharp distance peaks in the distribution ∼7 Å apart, almost symmetrically arranged around the predicted one (Figure 7A and Table 2). The shorter distance had lower amplitude compared to the longer distance. (Figure 7A and Table 2). We speculated that the two distances could have arisen from two distinct conformations of the spin label or of the protein. The ‘snuggly fit’ option of MtsslWizard (47) identifies two pockets around C193R1 (Supplementary Figure S9A) and this replicates the observed trace (Supplementary Figure S9B), suggesting that the distances reflect two preferred conformations of the spin label and not of the protein.

**Table 2.** Summary of the main distances of XPD in different binding states measured by PELDOR; the predicted values are shown in the legend

Mutant	Apo	ATPγS	ADP^.^AlFx	ADP	AMP–PNP	ssDNA	bDNA*	AMP–PNP and ssDNA*	AMP–PNP and bDNA*	ADP and ssDNA*
D13-N607	46.6	/	46.4	46.6	46.6	46.6	/	46.6	/	46.6
E122-Y306	32.3/40 (1:0.2)	/	/	/	/	/	32.3/40 (1:0.2)	32.9/40 (1:0.3)	33/40 (1:0.2)	/
A193-Y306^1^	28.3/35.6 (0.6:1)	28.3/35.6 (1:0.3)	27.4/35.9 (1:0.3)	27.2/35 (1:0.4)	27.8/36.1 (1:0.7)	27.8/35.6 (0.8:1)	28.3/35.6 (0.7:1)	27.8/35.6 (1:0.9)	/	/
Y306-D434	70.5	/	/	/	/	/	71.2	71.8	71.2	/
S267-D434	48.4	/	/	/	/	/	/	49.7	/	/

All distances are expressed in Å; *ssDNA: single stranded DNA; bDNA: bubble DNA; hDNA: hairpin DNA; The MtsslWizard simulated main distances are: 47.5 Å (13R1–607R1), 33 Å (122R1–306R1), 31.5 Å (193R1–306R1), 72 Å (306R1–434R1) and 50 Å (267R1–434R1). ^1^ The simulated distances (MtsslWizard) are: 31.5 Å (unrestricted search of conformers) and 28.2/35.2 Å (snuggly fit); In case of two distances, the ratio between them is shown in brackets.

### Effect of nucleotide binding on XPD conformation

The cysteine mutant in HD1 (C13) is close to the Q-motif (Q8), which is involved in ATP binding and is well positioned to report on nucleotide binding. Neither AMP–PNP, ADP nor ADP-AlFx (which should mimic the transition state during ATP hydrolysis) changed the distance distribution between the motor domains (Figure 6B and Table 2) beyond a slight narrowing, most probably as a result of reduced flexibility. In the case of the Arch–HD1 distance, a significant change in the relative heights of the distribution was obtained in the presence of nucleotides (ADP, ATPγS, ADP-AlFx or AMP–PNP), the short distance becoming more populated than the long one (Figure 7A–D). The main distances however did not change significantly (<1 Å) (Figure 7A–D and Table 2). This suggests that nucleotide binding may induce subtle changes in protein side chain conformations that in turn affect the relative proportions of the spin label adopting alternative positions.

### Effect of DNA binding on XPD conformations

Binding of either ssDNA or bubble DNA did not significantly change the distance distributions of HD1–HD2, Arch–FeS or Arch–HD2 pairs, nor did subsequent addition of nucleotide (Figures 6C and 8, Table 2). The proportion of the longer distance (shoulder) in the case of Arch–FeS did not change in the presence of DNA (Figure 8B versus 8A and Table 2). In the case of Arch–HD2, the slight increase of ∼1 Å observed in both double mutants was in agreement with the crystal structure containing the 4-nt DNA (Figure 8D versus 8C and 8F versus 8E, Table 2). For the Arch–HD1 pair, there were only minor changes in the distribution of the two distances in the presence of either ssDNA or bubble DNA (Figure 7E–G).

In summary, the only changes in distance distributions that we observed for XPD were those locally induced by nucleotides and (to a lesser extent) by DNA in case of the Arch–HD1 pair, an observation we take to mean the protein is rigid.

## DISCUSSION

A detailed understanding of the conformational changes that accompany the molecular mechanisms of helicases remains a work in progress. Structural biology methods represent a valuable tool, each of them having advantages and disadvantages. The use of PELDOR to characterize the conformational states of proteins as a tool complimentary to crystal structures is increasingly established (51–55). The main advantages of the technique are: its precision, no limitations on protein size and the fact that the spin label generally used (MTSSL) is much simpler and smaller when compared with fluorescent labels. A simple plugin to the freely available PYMOL program, MtsslWizard, provides a straightforward method of predicting the distance distributions (47,48). PELDOR has not been applied yet to the investigation of structural changes that accompany SF1 or SF2 helicases in different binding states. Here we have applied PELDOR to characterize the conformational changes that occur within two classes of helicases: the well studied PcrA, which belongs to the SF1A family and the less well studied XPD, a member of the SF2B family.

### ATP binding brings domain 1A closer to 2A and 2B in PcrA

Crystal structures of soaked PcrA–AMMPNP complexes did not detect significant motion of the motor domains (1A, 2A) toward each other on nucleotide binding (50). However, this movement was clearly observed by PELDOR on addition of AMP–PNP, resulting in a movement of 4 Å (well outside experimental uncertainty). Such movement has been assumed to occur but soaking ligands into crystals can, as apparently is the case here, freeze out motions. This interdomain movement required the γ-phosphate of the nucleotide, as ADP addition resulted in a 1 Å increase in the separation of the domains (Figure 2B). Therefore, as expected, ADP and AMP–PNP had different effects on the relative conformation of the motor domains.

With AMP–PNP binding, we observed that domains 1A and 2B moved closer together (Figure 3B and Table 1), but there was no change in the distance separating 2A and 2B (Figure 4B and Table 1). We are thus able to identify that domain 1A moves independently of domains 2A and 2B in the absence of DNA. Such a motion of domain 1A was recently proposed for *Deinococcus radiodurans* UvrD (56) and more generally for SF1A helicases (49).

### DNA binding induces movement of domain 2B and rigidifies the motor domains

In PcrA (and related proteins) domain 2B closes through a swivelling of ∼160° around a hinge region when the protein binds to a 3′-ss/dsDNA junction. We show here that PELDOR can successfully monitor the conformational changes induced by dsDNA. Surprisingly the PELDOR data suggest that dsDNA with a 3′ ssDNA tail does not shift the conformation entirely to the ‘closed’ structure, rather the ‘open’ conformation remains visible (Figures 3C, D and 4C, D). The heterogeneity could be due to incomplete DNA binding at the high salt concentration necessary to stabilize the protein, or may reflect mixed DNA-bound populations with differing conformations in solution as seen in FRET studies at low salt concentrations for UvrD bound to a partially single-stranded duplex DNA (8). Our PELDOR data also point to rigidification of the motor domains when DNA binds, an observation often not possible from a crystal structure. Furthermore we observe that DNA binding acts synergistically with nucleotide triphosphate in tightening the gap between the two motor domains (Figure 2D).

Although both the predicted and the experimental data follow the domains movements in the ‘product’ and ‘substrate’ complexes, the motions differ in some cases by 4–7 Å (Figures 2, 3 and 4, Table 1). These differences seem to have two contributions:

- DNA is reported by the crystal structures but not by the PELDOR data to affect the position of domain 2A relative to 1A and 2B (the same difference of ∼5 Å between the predicted and experimental distance for 1A–2A and 2A–2B, see Figures 2C, D, and 4C);
- AMP–PNP is reported by the crystal structures but not by the PELDOR data to affect the position of domain 2B relative to 1A and 2A (the predicted distances of both 1A–2B and 2A–2B show a difference of ∼5 Å between DNA and DNA + AMP–PNP).

These small discrepancies might be due to the different solution conditions used in our study compared with those of crystallization (e.g. ∼500 mM salt in our study versus 100 mM or lower in the crystal solution, the three extra nucleotides of the 3′-overhang in our DNA etc.). Alternatively, it is possible that the crystal packing may have trapped one particular protein conformation. Figures 2, 3 and 4 show that the width of the distance distribution varies between different complexes, suggesting the protein is dynamic.

In conclusion, PELDOR detects conformational changes of PcrA domains that are consistent with its helicase activity: in the absence of DNA, the equilibrium between the ‘open’ and the ‘closed’ state of domain 2B is shifted toward the former, at least at high salt concentration. When the protein binds the DNA duplex, the 2B domain adopts the ‘closed’ conformation, which was recently shown to represent the active state of the UvrD helicase (28). DNA binding also reduces the flexibility between the two motor domains. Nucleotide triphosphate binding brings domain 1A closer to 2A and 2B, regardless of the presence or absence of DNA.

### XPD helicase

While the PELDOR analysis of PcrA revealed conformational changes when binding DNA and/or nucleotide, XPD appears to be much less dynamic and in fact rigid. We did not detect any major conformational changes, in the presence of neither nucleotide nor DNA. Although somewhat surprising, the lack of conformational changes observed with XPD upon nucleotide or DNA binding are not unprecedented. The SF2 helicase Hel308 shows very little change in the relative organization of the motor domains between the apo, ADP and AMP–PNP bound forms (57). One caveat is that the nucleotides were soaked into the crystals, which could have prevented the reorganization of the motor domains. More recently, the structures of the CRISPR associated Cas3 helicase crystallized in the presence and absence of dATP showed only subtle changes in motif conformations with no overall gross changes in domain organization (58). The current model predicts that the translocated ssDNA strand passes through the central pore formed by the HD1, 4FeS and Arch domains (33–35,37,38). In the context of NER, where XPD binds to internal sites on ssDNA and unwinds bubbles, the DNA cannot thread through the pore starting from the terminus; this requires the Arch domain or the 4FeS domain to move (59). Our PELDOR data do not report clear evidence of a more open conformation of the 4FeS–Arch and HD1–Arch domain pairs. The only evidence of a more open structure is the shoulder corresponding to a longer distance between the 4FeS domain and the Arch domain of TaXPD (122R1–306R1). The DeerAnalysis program validates this as a real distance and not an artefact (Supplementary Figure S10). The difference between the two distances is only 6–7 Å, so this may not represent a fully open structure that permits DNA to pass through. However, this state is 5× less populated than the ‘closed’ state (observed in crystal structures), which would be consistent with it having a shorter lifetime (42). The distance distributions do not significantly change upon addition of DNA. This would be in agreement with a study that reports DNA binding favors neither the closed nor an as yet hypothetical open conformation (42). An alternative explanation is that the shoulder arises from different favoured spin label conformations. We tested for this using the snuggly fit function of MtsslWizard, however, we could not identify any combination of parameters that accurately reported on both distances. In conclusion, the interpretation of this shoulder as a reflection of a mechanistically-relevant XPD conformation remains plausible, but should be treated with care.

The apparent immobility of the Arch and/or 4FeS domain led us to weaken the contact between them by introducing two further mutations in the Arch domain, V324A (part of a hydrophobic interface with the 4FeS domain) and F326E (close to E103 and E107 of the 4FeS domain) (Supplementary Figure S11). The expression of this construct was very poor and the yellow-brown color characteristic of the 4Fe-4S cluster was rapidly lost. Since the amino acid changes do not reside in the 4FeS domain we interpreted the result as an indication that the interaction with the Arch domain stabilizes the 4Fe-4S cluster. This suggests that any opening of a channel between the Arch and FeS domains would need to be very short-lived *in vivo*.

### Concluding remarks

PELDOR has proven to be a very useful technique for the measurement of accurate distances between defined protein side chains (51–55). The high precision of PELDOR, coupled with the requirement for only one type of label, represent significant advantages over fluorescence techniques. Here, we have shown that PELDOR can be applied to the study of conformational changes in the reaction cycles of SF1 and SF2 helicases, yielding information not observed in X-ray crystallography. In some cases of PcrA we observed mixed populations occupying alternative protein conformational states highlighting the dynamic nature of the SF1 family. The technique thus holds great promise as a method to investigate the diverse family of RNA and DNA helicases where cyclical conformational change is fundamental to protein function.

## Supplementary Material

SUPPLEMENTARY DATA
