# Supplementary material for: Taking a molecular motor for a spin: helicase mechanism studied by spin labeling and PELDOR
Source: Nucleic Acids Res. 2015 Dec 10;44(2):954–68. doi: 10.1093/nar/gkv1373 (PMC4737156; doi:10.1093/nar/gkv1373)
Supplement: SUPPLEMENTARY DATA [file supp_44_2_954__index.html]

Taking a molecular motor for a spin: helicase mechanism studied by spin labeling and PELDOR — Taking a molecular motor for a spin: helicase mechanism studied by spin labeling and PELDOR — SUPPLEMENTARY DATA 

# Taking a molecular motor for a spin: helicase mechanism studied by spin labeling and PELDOR

## SUPPLEMENTARY DATA

- SUPPLEMENTARY DATA
