# Supplementary material for: Taking a molecular motor for a spin: helicase mechanism studied by spin labeling and PELDOR
Source: Nucleic Acids Res. 2015 Dec 10;44(2):954–68. doi: 10.1093/nar/gkv1373 (PMC4737156; doi:10.1093/nar/gkv1373)
Supplement: SUPPLEMENTARY DATA [file supp_gkv1373_nar-03080-f-2015-File010.pdf]

Supporting material for:

Taking a molecular motor for a spin: helicase mechanism studied by spin labelling and PELDOR

Diana Constantinescu Aruxandei[1], Biljana Petrovic-Stojanovska[1], Olav Schiemann[2], James H. Naismith[1] and Malcolm F. White[1]

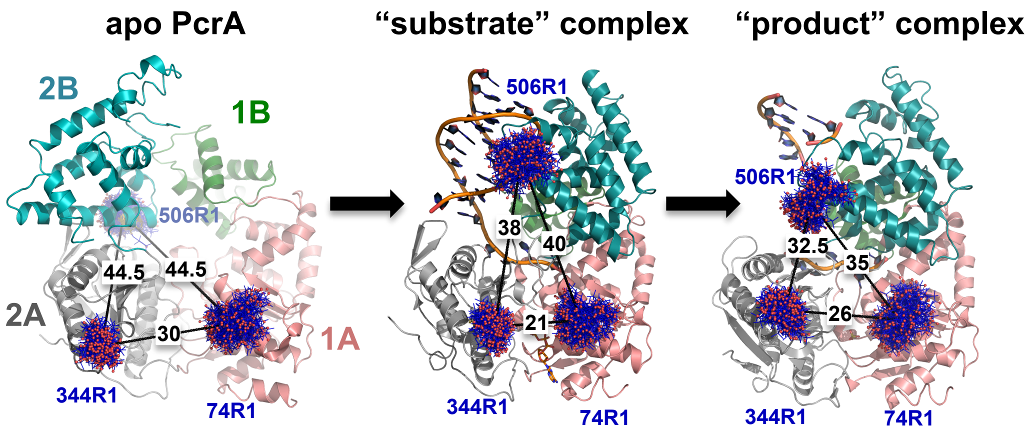

Figure S1. Spin label conformational distributions (blue-red sticks) simulated with MtsslWizard for apo state (PDB: 1pjr), "substrate" complex (PDB: 3pjr) and "product" complex (PDB: 2pjr) of PcrA. The main simulated distances (Å) between the spin labels for the cysteine pairs between specific domains are shown in black: 74R1 (1A) – 344R1 (2A), 74R1 (1A) – 506R1 (2B) and 344R1 (2A) – 506R1 (2B).

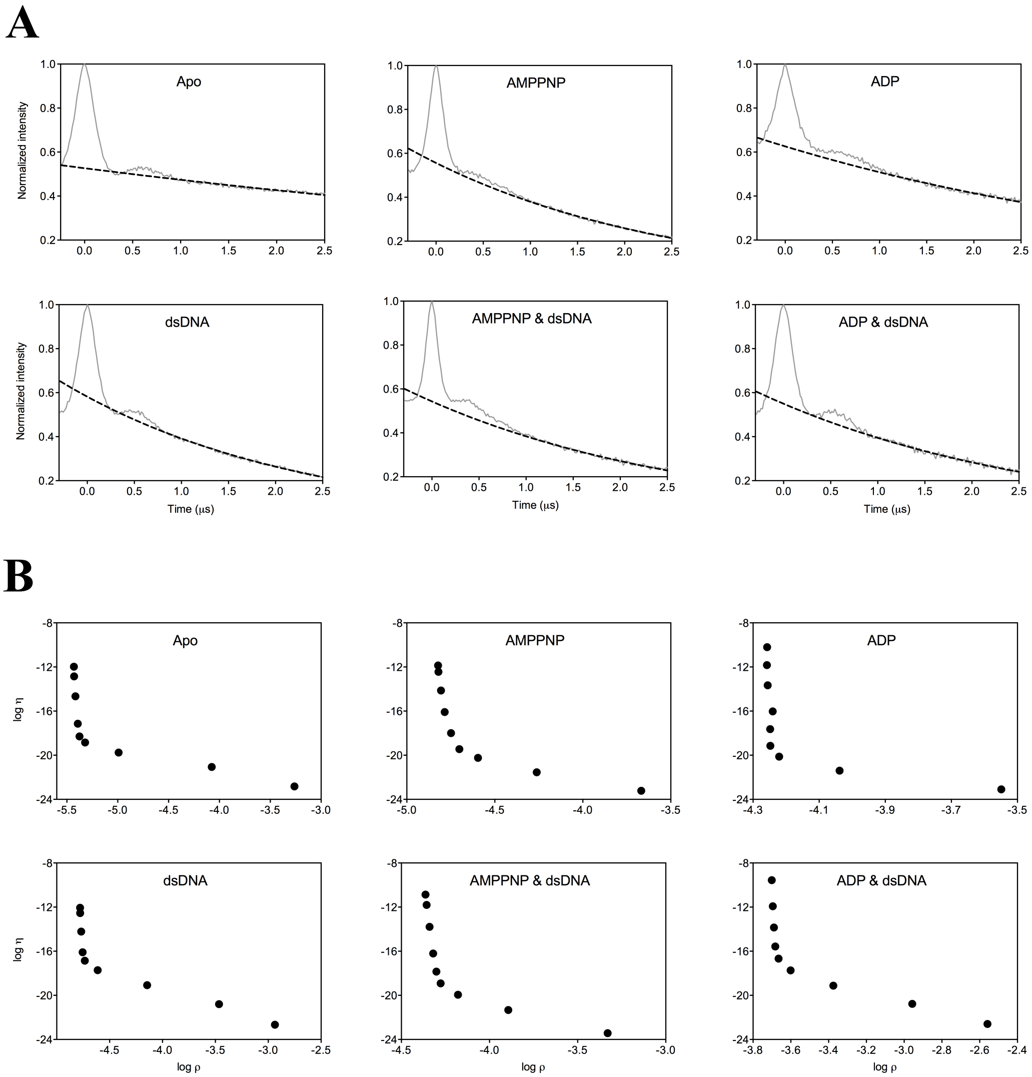

Figure S2. PELDOR data of PcrA 74R1 – 344R1. (A) Normalized raw PELDOR time traces (continuous lines) and applied background correction (dotted lines); (B) The corresponding L-curves.

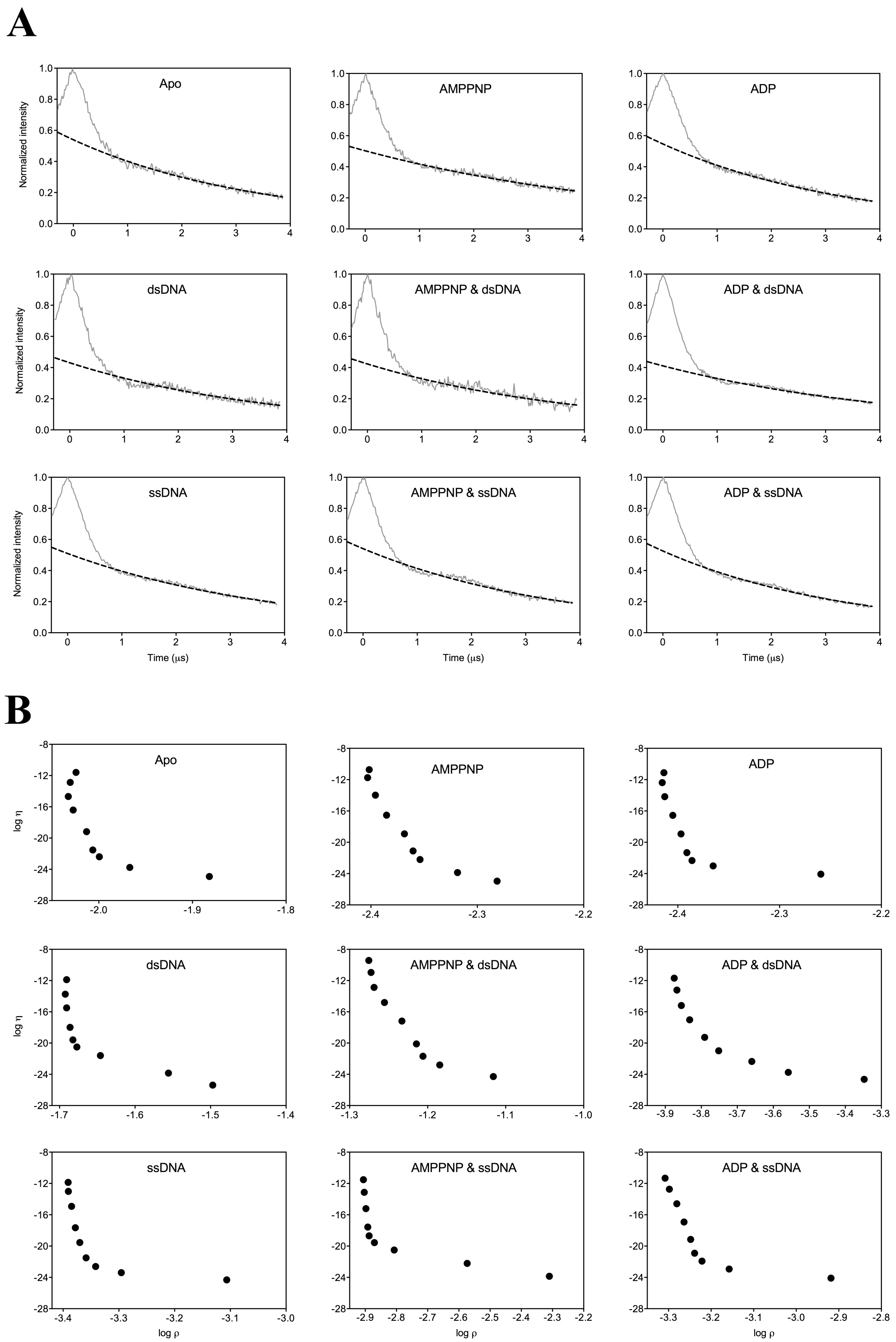

Figure S3. PELDOR data of PcrA 74R1 – 506R1. (A) Normalized raw PELDOR time traces (continuous lines) and applied background correction (dotted lines); (B) The corresponding L-curves.

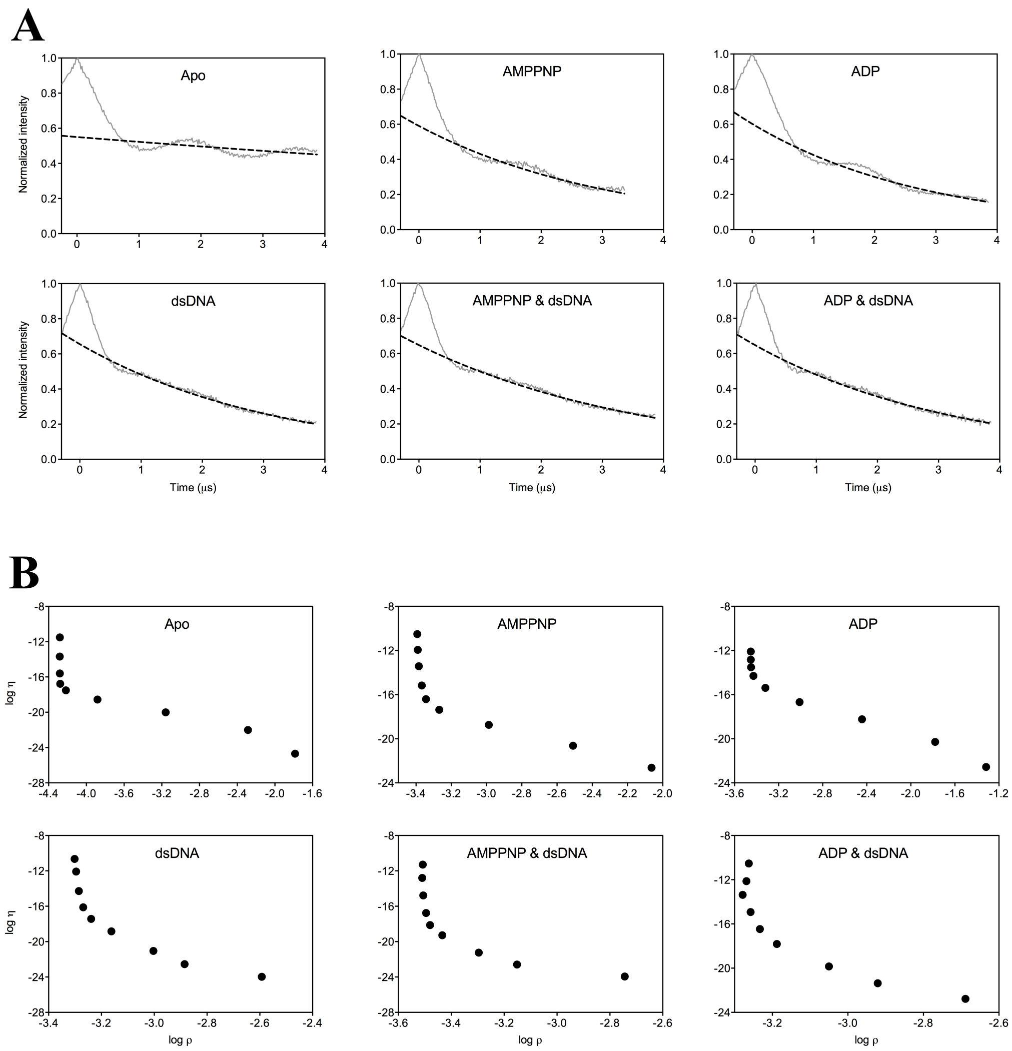

Figure S4. PELDOR data of PcrA 344R1 – 506R1. (A) Normalized raw PELDOR time traces (continuous lines) and applied background correction (dotted lines); (B) The corresponding L-curves.

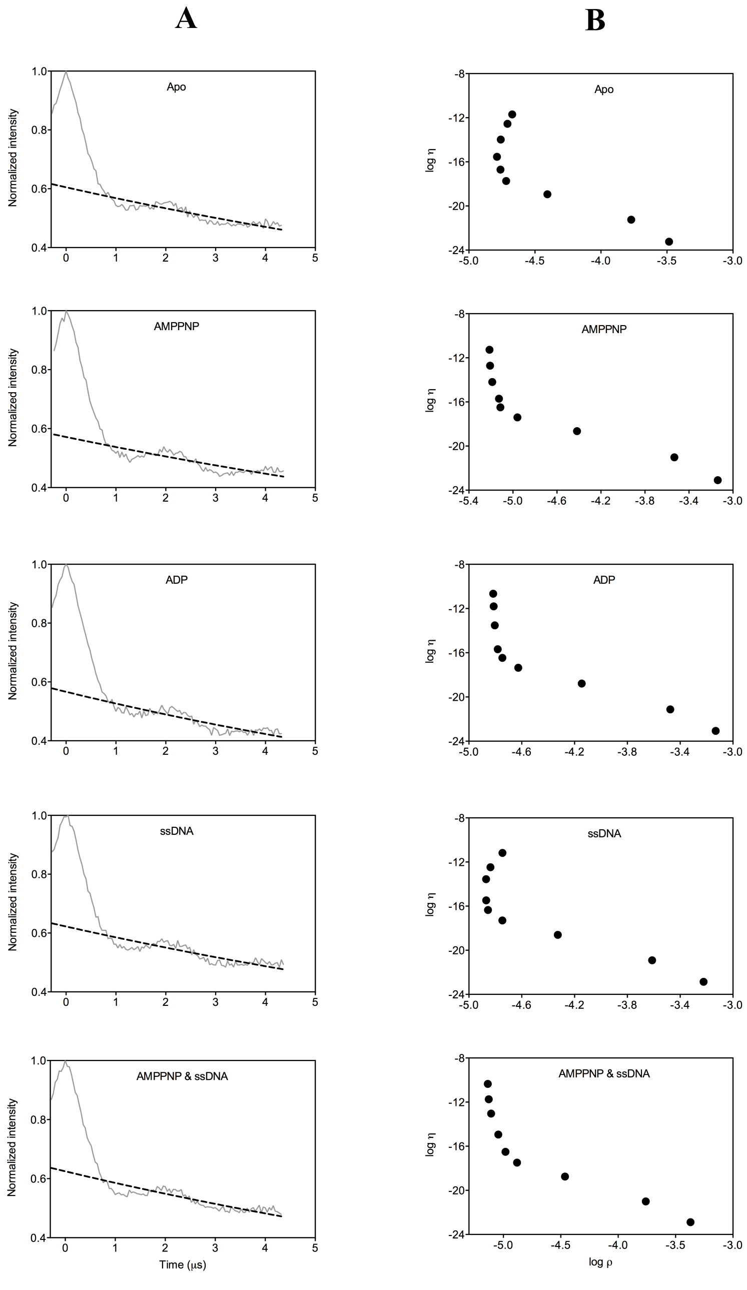

Figure S5. PELDOR data of TaXPD 13R1 – 607R1; (A) Normalized raw PELDOR time traces (continuous grey lines) and applied background correction (dotted black lines); (B) The corresponding L-curves.

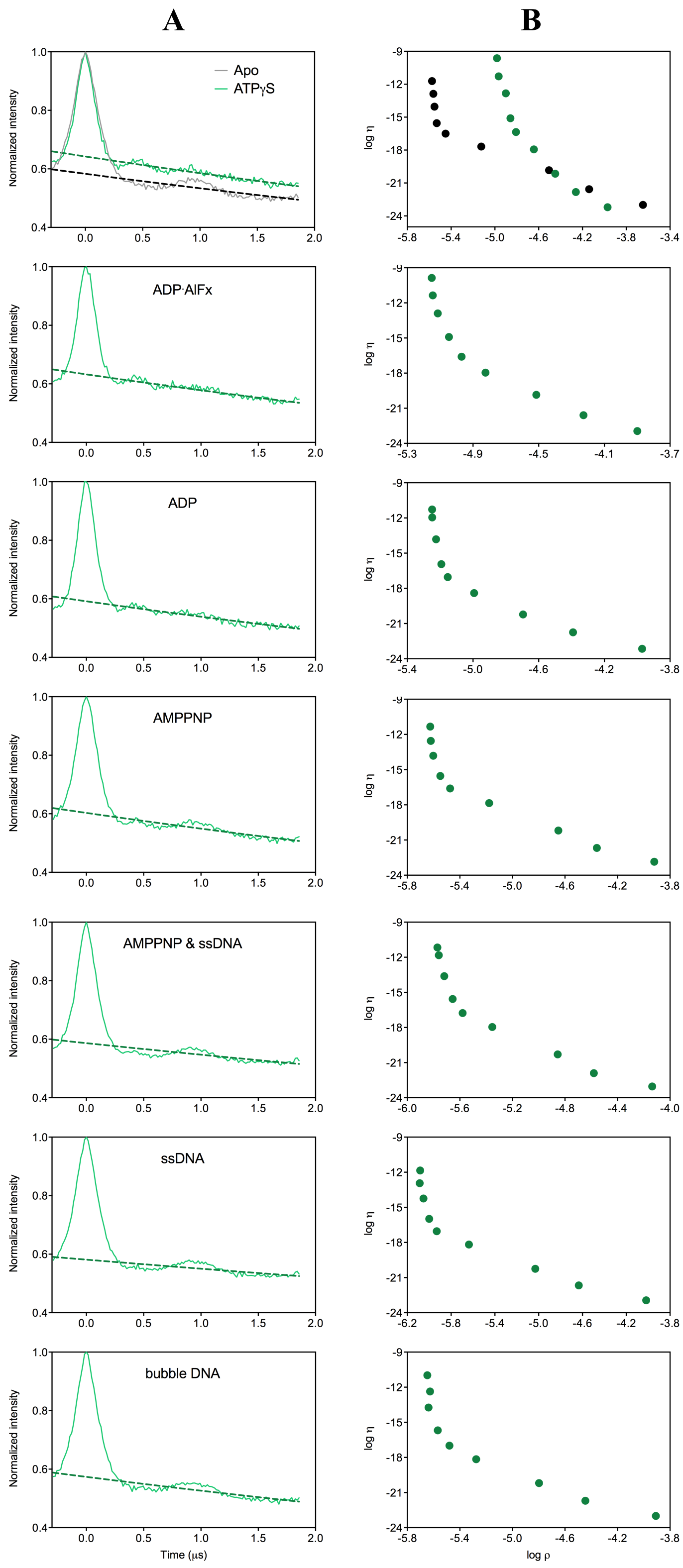

Figure S6. PELDOR data of TaXPD 193R1 – 306R1; (A) Normalized raw PELDOR time traces (continuous lines) and applied background correction (dotted lines); the black dotted line is the background for the grey time trace. (B) The corresponding L-curves.

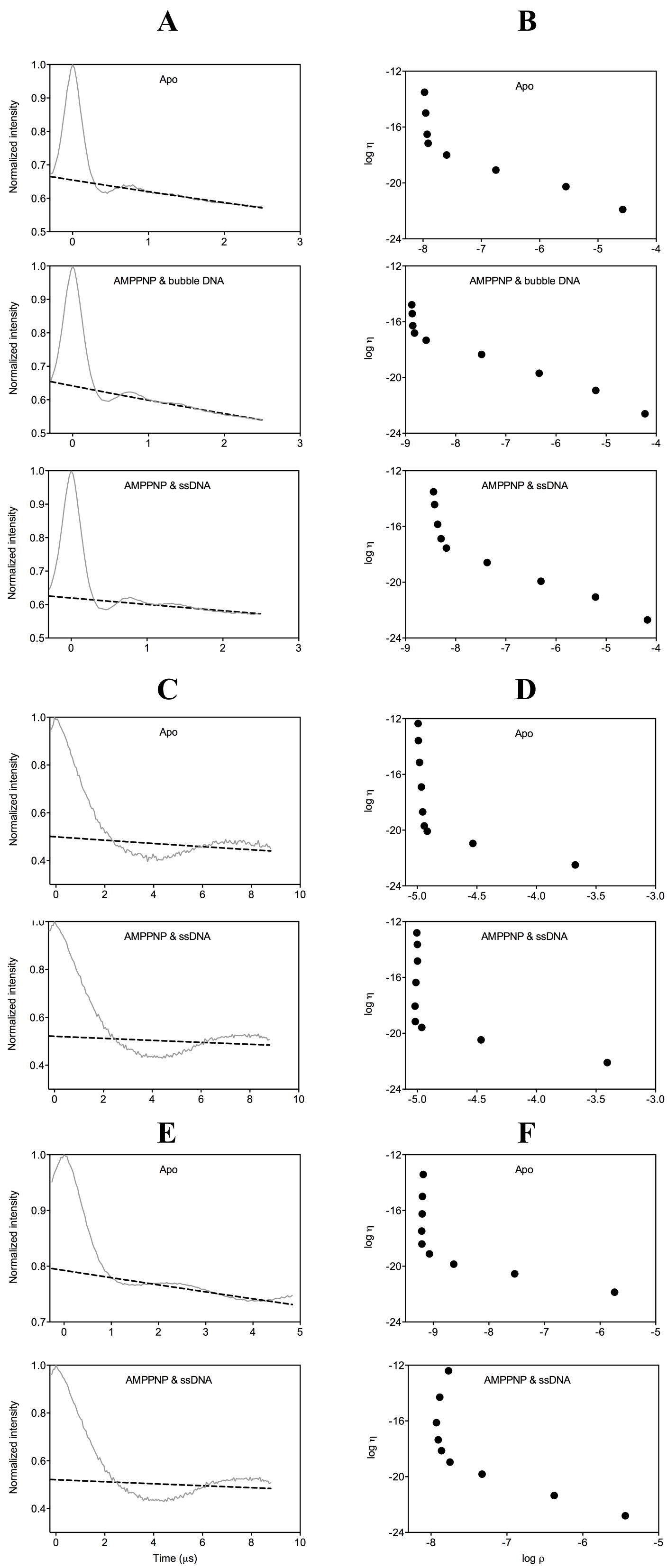

Figure S7. PELDOR data of TaXPD 122R1 – 306R1 (A, B), 306R1 – 434R1 (C, D) and 267R1 – 434R1 (E, F); (A, C, and E) Normalized raw PELDOR time traces (continuous grey lines) and applied background correction (dotted black lines); (B, D, and F) The corresponding L-curves.

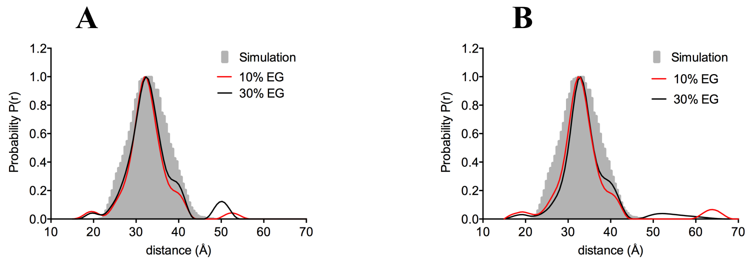

Figure S8. Tikhonov derived distance distributions of apo XPD (A) and XPD + AMPPNP & bubble DNA (B) in presence of either 10% (red lines) or 30% EG (black lines), compared to the simulated distance (grey shape. PDB 4a15).

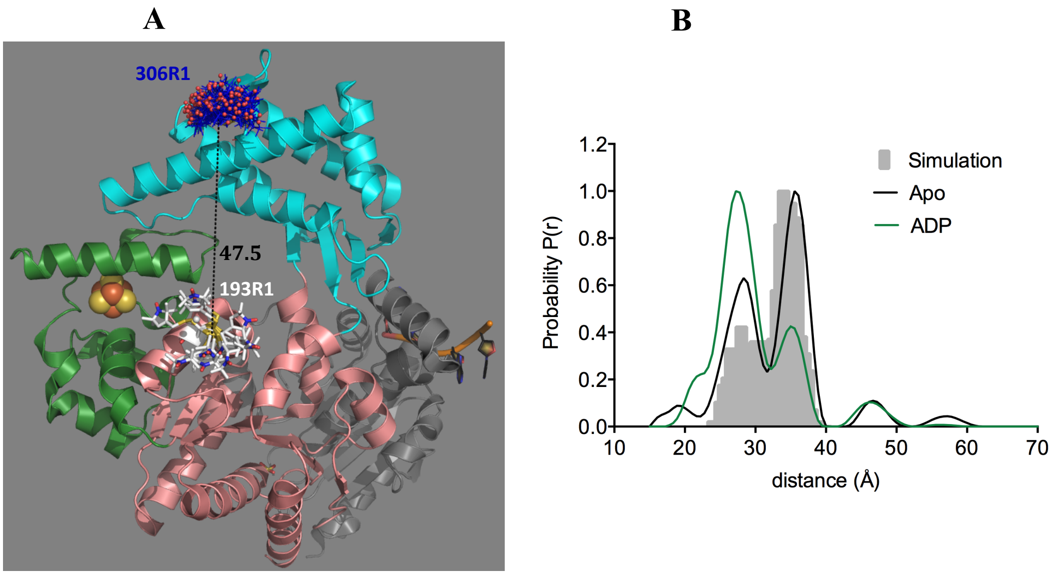

Figure S9. (A) Spin label conformational distribution simulated with MtsslWizard (PDB: 4a15) for 193R1 – 306R1; 193R1 (white sticks) was simulated using the snuggly fit option, with a vdW cutoff of 2.2 Å; the vdW cutoff for 306R1 was 3.4 Å. The domains are colored salmon (HD1), grey (HD2), green (4FeS domain) and cyan (Arch domain). The average simulated distance (Å) between the spin labels is shown in black. (B) Tikhonov derived distance distributions (black and green lines) in comparison with model derived distance distribution obtained from the simulation in (A) (grey shape).

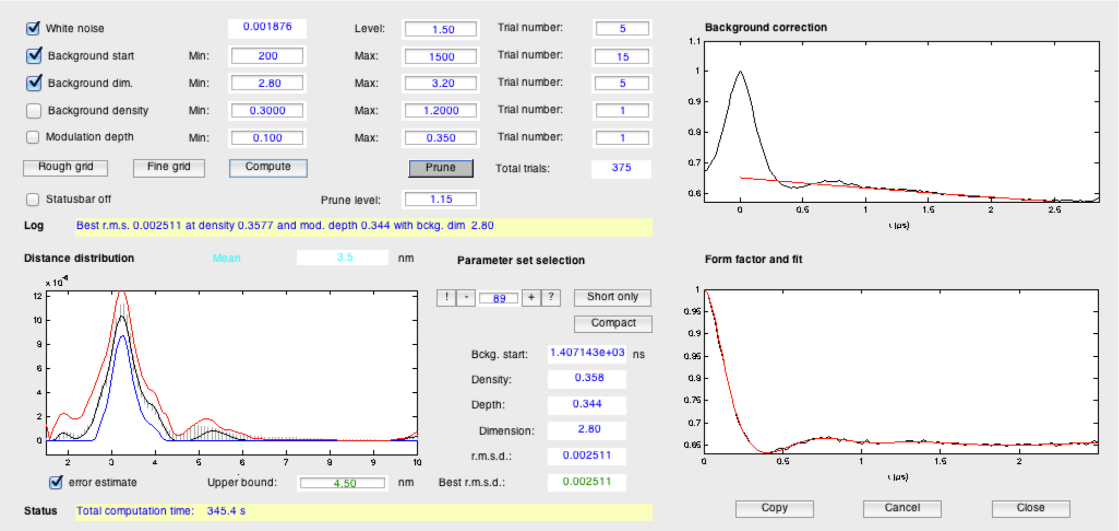

Figure S10. Screenshot of the validation window for XPD 122R1 – 306R1. The influences of noise and of uncertainties in background parameters were combined to obtain a total error estimate. The left panel shows the distance distribution with the best r.m.s.d. (black continuous line) and grey error bars (full variation of the probability of a given distance over all trials), a lower error estimate (blue continuous line) and an upper error estimate (red continuous line). The most populated distance together (~ 32 Å) with the shoulder (~ 40 Å) show little variation with the parameters, an indication of real distances. The position and the amplitude of the longest distance (~ 50-60 Å) vary significantly, suggesting that this distance is an artefact.

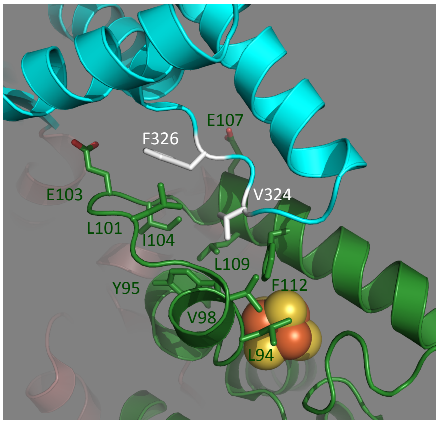

Figure S11. Close-up view of the interface between the Arch domain (cyan) and the 4FeS domain (green). The interface is mainly stabilized through hydrophobic interactions between V324 and F326 in the Arch domain and several residues in the 4FeS domain. To disrupt the interface V324 was mutated to alanine and F326 to glutamic acid.

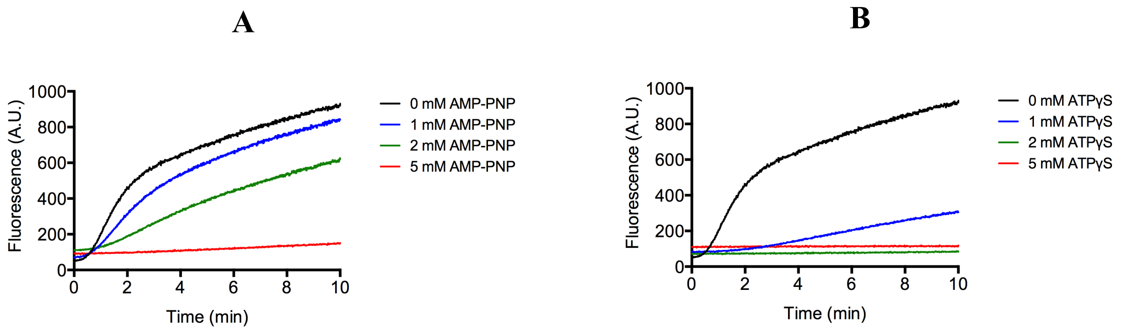

Figure S12. TaXPD helicase activity in the presence of ATP analogues. The helicase activity of TaXPD was assayed using a continuous fluorescence assay. TaXPD was incubated with AMP-PNP (A) or ATPγS (B) at the indicated concentrations in helicase buffer, as described in the methods. The reaction was initiated by the addition of 1 mM ATP and followed for 10 min. A concentration dependent inhibition of helicase activity was observed for both AMP-PNP and ATPγS, with the latter inhibitory at lower concentrations. This confirms that both analogues can bind to TaXPD.
